# Endovascular Repair of Arterial Iliac Vessel Wall Lesions with a Self-Expandable Nitinol Stent Graft System

**DOI:** 10.1371/journal.pone.0103980

**Published:** 2014-08-13

**Authors:** Birger Mensel, Jens-Peter Kühn, Andreas Hoene, Norbert Hosten, Ralf Puls

**Affiliations:** 1 Institute of Diagnostic Radiology and Neuroradiology, University Medicine Greifswald, Greifswald, Germany; 2 Department of Vascular Surgery, University Medicine Greifswald, Greifswald, Germany; 3 Diagnostic and Interventional Radiology and Neuroradiology, Helios Hospital Erfurt, Erfurt, Germany; University of Washington School of Medicine, United States of America

## Abstract

**Objective:**

To assess the therapeutic outcome after endovascular repair of iliac arterial lesions (IALs) using a self-expandable Nitinol stent graft system.

**Methods:**

Between July 2006 and March 2013, 16 patients (13 males, mean age: 68 years) with a self-expandable Nitinol stent graft. A total of 19 lesions were treated: nine true aneurysms, two anastomotic aneurysms, two dissections, one arteriovenous fistula, two type 1B endoleaks after endovascular aneurysm repair, one pseudoaneurysm, and two perforations after angioplasty. Pre-, intra-, and postinterventional imaging studies and the medical records were analyzed for technical and clinical success and postinterventional complications.

**Results:**

The primary technical and clinical success rate was 81.3% (13/16 patients) and 75.0% (12/16), respectively. Two patients had technical failure due to persistent type 1A endoleak and another patient due to acute stent graft thrombosis. One patient showed severe stent graft kinking on the first postinterventional day. In two patients, a second intervention was performed. The secondary technical and clinical success rate was 87.5% (14/16) and 93.8% (15/16). The minor complication rate was 6.3% (patient with painful hematoma at the access site). The major complication rate was 6.3% (patient with ipsilateral deep vein thrombosis). During median follow-up of 22.4 months, an infection of the aneurysm sac in one patient and a stent graft thrombosis in another patient were observed.

**Conclusion:**

Endovascular repair of various IALs with a self-expandable Nitinol stent graft is safe and effective.

## Introduction

Iliac Arterial Lesions (IALs) are caused by various pathologies, including aneurysms, dissections, arteriovenous (AV) fistulas, pseudoaneurysms, and traumatic or atraumatic perforations [1], [2], [3], [4]. The prevalence of solitary iliac artery aneurysms (IAAs) is approx. 0.03% and rare compared to aortic aneurysms [1], [5]. The frequency of other IALs is unknown, but their occasional appearance in the literature suggests their reasonably low occurrence [3], [4], [6], [7].

IALs require treatment to prevent complications such as fatal arterial bleeding in subjects with aneurysms and perforations or high-output cardiac failure in subjects with an AV fistula. In addition, more specific comorbidities exist such as increased risk of thrombosis or emboli, compression syndromes, and abdominal pain [1], [3].

All lesions can potentially be treated by open surgery. This is a well-established and reliable treatment; however, it is a time-consuming major procedure and requires general anesthesia. Endovascular repair of iliac lesions with stent graft placement is an effective minimally invasive option for treating IALs [8], [9]. Several different models of stent grafts are currently available (balloon-expandable, self-expanding, limbs from aortic stent grafts) [1]. The self-expanding Fluency Plus stent graft (BARD Peripheral Vascular, Tempe, Arizona) is a versatile device for treating different lesions in different body regions [6], [10], [11].

The purpose of this study was to assess the therapeutic outcome after endovascular repair of various IALs with a self-expandable Nitinol stent graft.

## Materials and Methods

This retrospective study was approved by the institutional review board of Greifswald University Hospital (BB039/13). All included patients gave their written informed consent for endovascular treatment. All medical records and images used in this study were anonymized and deidentified prior to analysis.

### Patients

From July 2006 to March 2013, 16 consecutive patients (13 men, 3 women) underwent endovascular repair of various IALs and were enrolled in this retrospective study. The mean age was 67.9±4.9 years (range: 49–79 years). A total of 19 lesions were treated: nine true aneurysms, two anastomotic aneurysms, two dissections, one AV fistula, two type 1B endoleaks after endovascular aneurysm repair, one pseudoaneurysm due to tumor erosion (colorectal cancer), and two perforations after percutaneous angioplasty **(** **Tab. 1** **)**. Demographic and clinical data, medical reports as well as pre-, intrainterventional, and postinterventional images including follow-up studies were obtained from the electronic patient records or the picture archiving and communication system.

**Table 1:** 

Patient no.	age/sex	Type of lesion	Location/side	Size of IAL dxl mm	Stent graft dxl mm	Additional procedure/reason
1	62/m	dissecting aneurysm	CIA/right	21×64	13.5×120	stent in distal stent graft/stent graft kinking
2	75/m	sacculated aneurysm	CIA/left	26×65	9×100	-
3	73/m	fusiform aneurysm	CIA/right	34×46	13.5×80	-
		fusiform aneurysm	IIA/right	44×57	13.5×80	
4	67/m	fusiform anastomotic aneurysm after Iliaco-femoral bypass	proximal/left	26×33	10×80	-
5	74/m	fusiform aneurysm	CIA/left	22×36	13.5×80	-
		fusiform aneurysm	CIA/right	33×60	13.5×40	
		fusiform aneurysm	IIA/right	28×34	13.5×40	
6	73/f	type 1B endoleak*	iliac leg/left	-	12×80	-
7	49/f	arteriovenous fistula most likely due to arterial plaque rupture	CIA/right	11	13.5×40	-
8	79/m	fusiform aneurysm	IIA/left	70×68	13.5×100	coiling IIA/to prevent endoleak
9	56/m	acute dissection after iliaco-profundal bypass	CIA/right	-	13.5×40	2nd stent graft proximally[10×40]/type 1A endoleak
10	77/m	perforated fusiform aneurysm	IIA/left	69×73	12×80	coiling IIA/to prevent endoleak
11	72/m	pseudoaneurysm due to tumor erosion	IIA/left	-	12×80	coiling IIA/to prevent endoleak
12	73/m	fusiform anastomotic aneurysm	iliaco-femoral bypass/right	25×27	12×80	mechanical and pharmacological thrombolysis/stent graft thrombosis
13	74/m	fusiform aneurysm	CIA/right	35×59	12×80	coiling IIA/to prevent endoleak
14	60/f	perforation after PTA	EIA/left	-	8×40	-
15	71/m	type 1B endoleak*	Iliac leg/right	-	13.5×80	-
16	52/m	posttraumatic	EIA/left	-	12×80	-

CIA = common iliac artery, IIA = internal iliac artery, EIA = external iliac artery, d = diameter, l = length.

* = reperfused IIA aneurysm 3 days (patient no. 6) resp. 5 days (patient no. 15) after infrarenal endovascular aneurysm repair.

### Preinterventional imaging and indication

Fifteen of the 16 patients (the one exception, patient no. 14, required an emergency procedure) underwent preinterventional computed tomographic angiography (CTA) on an 8-row multidetector scanner (LightSpeed Ultra; GE Healthcare, Milwaukee, USA) using a standard protocol (slice thickness: 1.25 mm, pitch 1.35∶1). 120 ml of intravenous contrast agent (Imeron 350; BRACCO Imaging, Constance, Germany) was administered at a flow rate of 4.0 ml/s using bolus tracking. In selected indications, an additional venous-phase scan was performed. The standard protocol included coronal and sagittal reformations.

The decision for endovascular repair was made jointly by the interventional radiologist and the vascular surgeon. A patient was considered suitable for stent graft placement when safe coverage of the lesion (at least 1.5 cm proximally and distally) seemed to be achievable on the basis of preinterventional planning of the procedure using CTA and there were no contraindications to endovascular treatment (e.g., severe renal failure, manifest hyperthyroidism, known contrast medium allergy, coagulation disorder) [12]. The preinterventional CTA was used for size measurement of the treated vessels and lesions (baseline measurement).

### Endovascular repair

All patients underwent digital subtraction angiography (Axiom-Artis, Siemens Medical Solutions, Forchheim, Germany) with intra-arterial contrast agent injection (Imeron 300; BRACCO Imaging, Constance, Germany). After local anesthesia a percutaneous, ipsilateral access (7–10F introducer sheath) via the common femoral artery was used. Patient no. 7 received a bilateral access. In patients no. 1 and 13, an additional contralateral access (4F introducer sheath, 4F pigtail catheter) was used, enabling contrast injection during the implantation procedure. Before endovascular treatment, each patient received 5000 international units of heparin intra-arterially. All patients were treated with Fluency Plus stent grafts (BARD Peripheral Vascular, Tempe, Arizona) according to the instructions for use (IFU). After stent graft deployment, a balloon angioplasty was performed to affix of the stent graft along the vessel wall. To reduce the risk of endoleak proximal and distal neck oversizing of the stent graft by at least 10% was used. Depending on the underlying pathology, in case of a large-sized internal iliac artery or major side branches, embolization with fibered platinum coils (VortX 18 or 35, Boston Scientific Corporation, Natick, USA) was performed during the same intervention to prevent type 2 endoleaks.

Manual compression (10–15 minutes) was used for hemostasis of the access site, followed by compression dressing of the groin for 9–10 hours. For the first 48 hours after the procedure, patients were heparinized to achieve a target partial thromboplastin time of 50–60 seconds. All patients received antiplatelet therapy consisting of clopidogrel bisulfate (Plavix, Sanofi-Aventis Bridgewater, USA) at a dose of 75 mg per day for one month (after a 300 mg loading dose the day of the intervention) and 100 mg Aspirin (Bayer, Leverkusen, Germany) as lifetime medication.

### Follow-up

Patients underwent ultrasound or CTA (same protocol as for preinterventional imaging but including a venous-phase scan in all cases) and clinical examination at 1, 3, 6, and 12 months and then annually. Median follow-up time was 22.4 months (interquartile range: 7.0–29.5 months). The largest diameter of the aneurysm sac was measured at each follow-up examination. In case of IAA the size was determined at each follow-up. An increase in aneurysm greater than 20% compared to the baseline measurement was classified as complication.

### Definitions

The definitions of technical and clinical success are modified from the recommendations of the Ad Hoc Committee for Standardized Reporting Practices in Vascular Surgery of the Society for Vascular Surgery [13].

*Primary technical success* was defined as the successful deployment of one or more stent graft(s) within the intended artery/ies with elimination of the underlying pathology (e.g. aneurysm, dissection) without an endoleak, stent graft occlusion or immediate major complication (e.g. acute stent graft occlusion, dissection).

*Additional procedures* include other endovascular treatment to achieve primary technical success (e.g. coiling of internal iliac artery).

*Secondary technical success* was the need for a second endovascular procedure to achieve the above-mentioned aims.

*Primary clinical success* was the sustained solution of the underlying pathology without the need for secondary endovascular or surgical procedure during the same hospital stay confirmed by CTA.

*Secondary clinical success* was the need for a second endovascular procedure to achieve the above-mentioned aims or the spontaneous cessation of the clinical failure within 1 month after the last endovascular procedure (e.g. endoleak).

*Complications* were assessed within 30 days of the initial stent graft placement. They were classified as minor or major. Minor complications were all undesired events with no or nominal treatment (e.g. hematoma at access site) or recovered spontaneously. Major complications were defined as those that required an invasive treatment, led to hospitalization >24 h (e.g. stent graft occlusion treated by surgery) or death [13].

## Results

**Figure 1:**
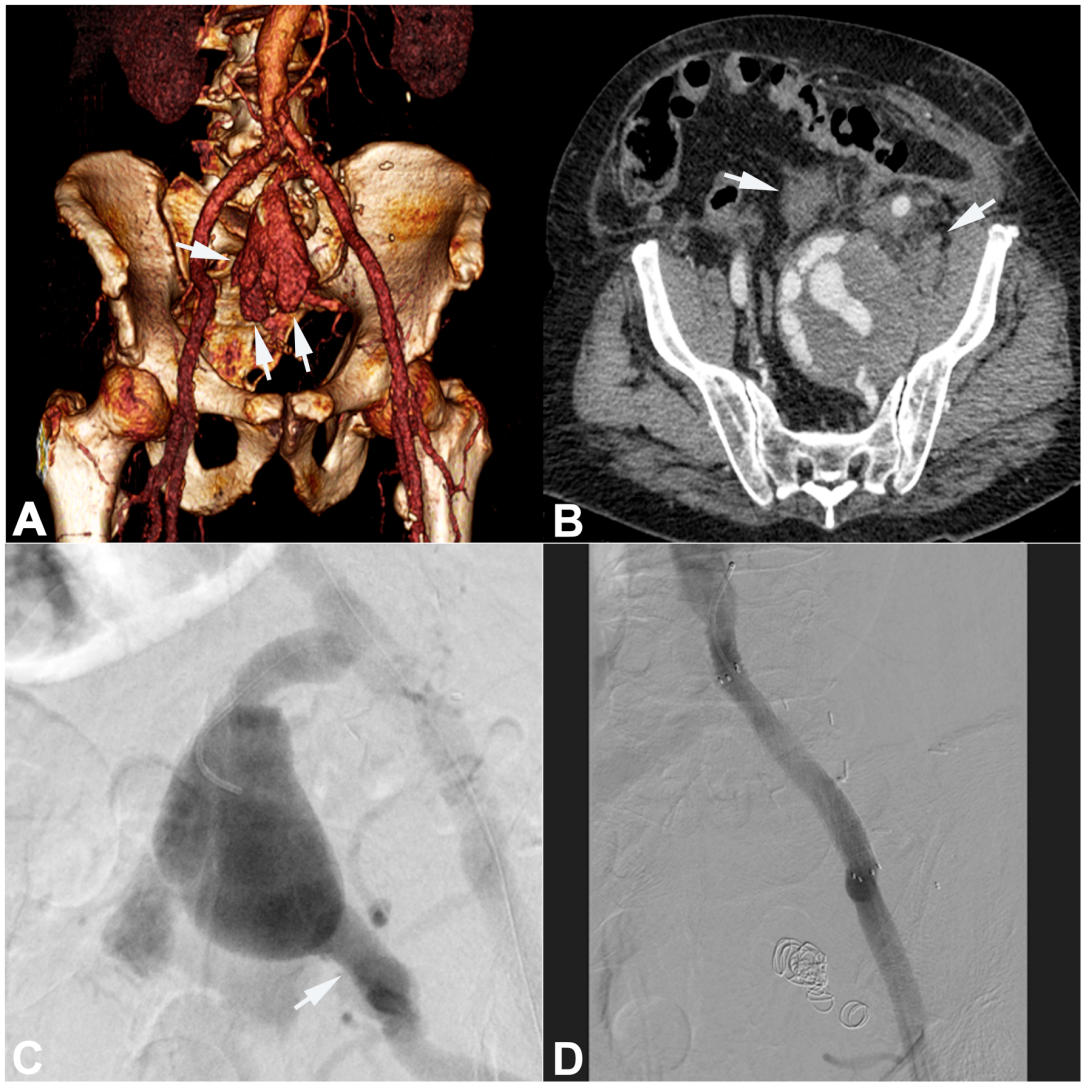
Successful stent graft placement of a ruptured IIA aneurysm in a 77-year-old man with acute abdominal pain in the left lower quadrant. **A**) Volume reconstruction (VR) shows a large left IIA aneurysm with the irregular boundaries (arrows) indicating a rupture. **B**) On axial CT scan the true size of the aneurysm is depicted with active bleeding within the thrombosed part and blood surrounding the aneurysm (arrows). **C**) Selective angiography shows a strong IIA aneurysm (arrow) with major side branches. **D**) Angiogram after stent graft placement and coiling of aneurysm shows complete exclusion of the aneurysm from blood flow.

A total of 16 patients with 19 IAL's were treated with 18 stent grafts. In seven patients (43.7%) an additional procedure was performed during the same intervention **(** **Fig. 1** **)**. An overview of all procedures is presented in Table 1 **(** **Tab. 1** **)**.

**Figure 2:**
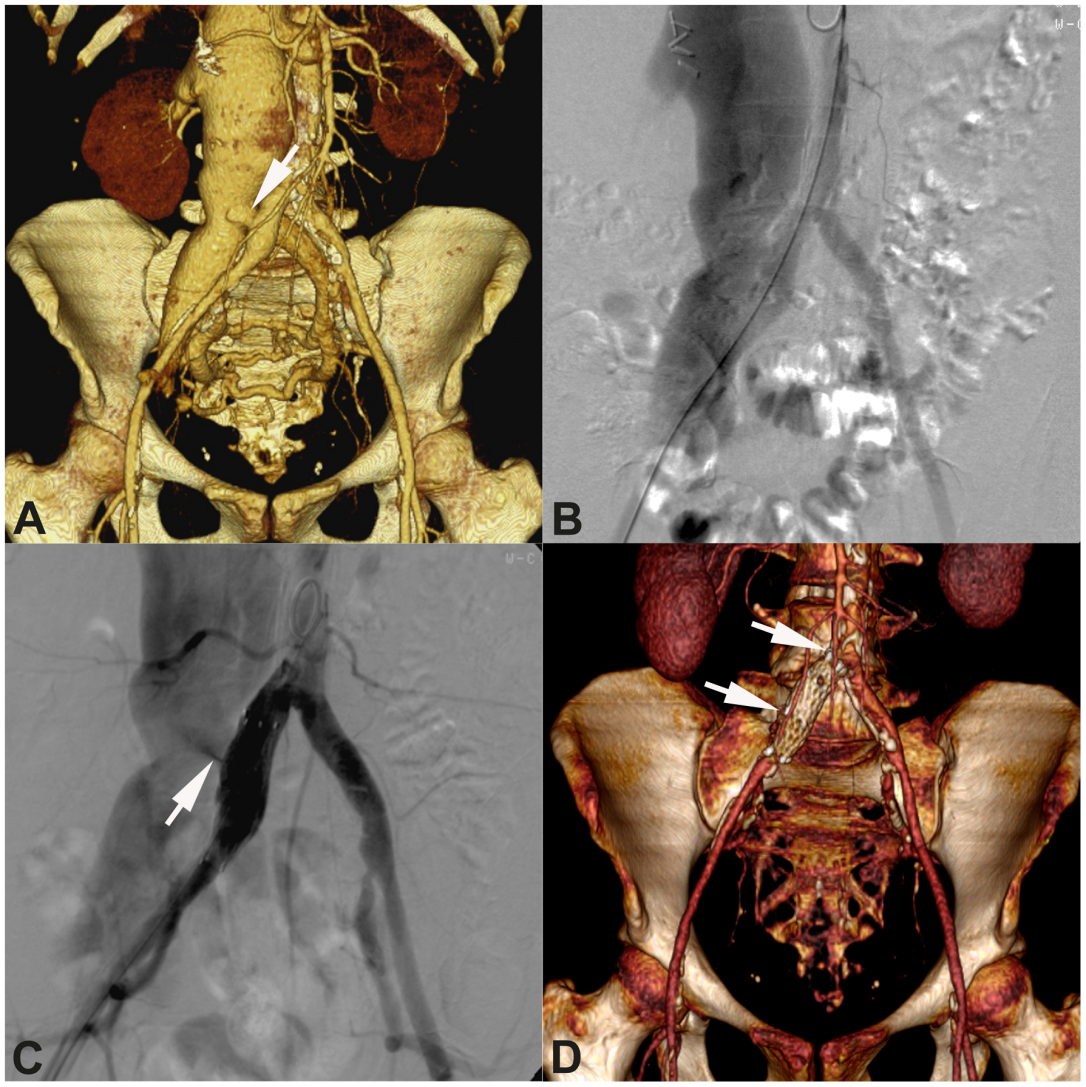
Successful endovascular repair of an iliac AV fistula in a 49-year-old woman with progressive dyspnea, right leg pain and edematous swelling of the extremity. **A**) Volume reconstruction (VR) with AV fistula between the right common iliac artery and vein (arrow); note the massive enlargement of the inferior cava vein. **B**) Angiographic correlation of the finding. **C**) The control angiogram after stent graft placement reveals a type 1A endoleak with persistent AV fistula (arrow). **D**) VR 12 months after implantation of a second stent graft (arrows) shows complete occlusion of the AV fistula.

The primary technical success rate was 81.3% (13/16 patients). Three patients (patients no. 5, 7 and 12) failed primary technical success due to type 1A endoleak, type 1A endoleak with persisting AV fistula, and acute stent graft thrombosis (12 h after the intervention). Patient no. 12 underwent surgical thrombectomy after failed endovascular thrombolysis. The primary clinical success rate was 75.0% (12/16). In one case, the intervention was classified as clinical failure because the patient developed severe stent graft kinking with significant flow reduction on the first postinterventional day (patient no. 1). This patient was treated by placing an additional stent within the first stent graft to straighten the kinked segment. In another case (patient no. 7), a stent graft extension became necessary **(** **Fig. 2** **)**.

The secondary technical success rate was 87.5% (14/16). Patient no. 5 showed spontaneous resolution of type 1A endoleak on the 20th postinterventional day. The secondary clinical success rate was 93.8% (15/16). None of the patients died within 30 days.

The minor complication rate was 6.3%. One patient (patient no. 3) developed a painful hematoma at the access site on the first postinterventional day. The patient was treated with analgesics and local cooling. The major complication rate was 6.3%. Patient no. 8 developed ipsilateral iliac vein thrombosis on the 26th postinterventional day, treated with immobilization and heparin.

Significant aneurysm growth in patients with treated IAA was not observed during follow-up. One patient was lost to follow up (patient no. 3).

Nine months after the intervention, patient no. 8 developed an infection of the aneurysm sac, requiring percutaneous drainage and intravenous antibiotics. Patient no. 9 showed thrombosis of the treated CIA 9 months after stent graft implantation. Therapy was not performed because of end stage oropharyngeal squamous cell carcinoma.

## Discussion

This study investigated the therapeutic outcome after endovascular repair of various IALs using a self-expandable Nitinol stent graft system. The primary and secondary technical success rate was 81.3% and 87.5%, respectively. The primary and secondary clinical success rate was 75.0% and 93.8%. The minor and major complication rate was 6.3%.

In previous studies of endovascular management of IALs, several different stent grafts with various architecture and deployment mechanisms were used for treating the same kind of IAL (e.g. aneurysm) [4], [8], [14], [15]. In contrast, this study focuses on patients with various IALs treated with the same stent graft. The Fluency Plus stent graft is a self-expanding Nitinol stent encapsulated in expanded polytetrafluoroethylene (according to IFU). Available data suggest that the stent graft has an extensive therapeutic spectrum with encouraging results [6], [10], [11].

Isolated IALs caused by different pathologies are relatively rare [2], [3], [4], [5]. The most widely investigated lesions in terms of epidemiology, treatment options and outcome are IAAs [1], [12]. Some investigations show that elective endovascular repair of isolated IAAs is safe and effective and is associated with decreased intraoperative blood loss, shorter hospital stay, and lower mortality compared with open surgery [8], [9]. The technical success of endovascular repair is about 100% with primary patency rates at 3 years from 86% to 97% [9], [14], [16]. In the 9 (true) aneurysms treated in this study, the technical success rate was 90%. One patient had a persistent small type 1A endoleak, which thrombosed spontaneously on the 20th postinterventional day. One patient with a type IIA aneurysm developed iliac vein thrombosis and later on an infection of the aneurysm sac. Compression of adjacent structures with subsequent development of deep vein thrombosis is known but its occurrence after endovascular interventions appears to be rare [1], [17]. Late aneurysm sac infection is absolutely uncommon.

For endovascular repair of most of the other lesions treated in this study, only limited data exist. Ludwig et al. reported a successful endovascular repair of an iliac AV fistula following a plaque rupture of the CIA in a 78-year-old man with pulmonary embolism [3]. Sanada et al. successfully treated two patients with endovascular stent grafting after pseudoaneurysm formation of the iliac arteries following local infection after rectum amputation and a psoas abscess [4]. Akpinar et al. reported a 36-year-old woman with acute arterial bleeding after lumbar laminectomy. She was successfully treated with stent grafting. However, the follow-up time did not exceed 12 months. The evaluation of long-term patency is crucial for the anchorage of the stent graft in IAL. In our study, only one patient suffering from end stage cancer developed stent graft thrombosis during follow-up, supporting the reported long-term patency of iliac stent grafts [14], [16].

The median follow-up time in our study was only 22.4 months; therefore, further studies presenting long-term results are necessary. Other limitations are the small number of patients, the use of either CTA or ultrasound for follow-up, and the retrospective design of this single-center study. Unlike stent grafts from other manufacturers, Fluency Plus stent grafts are only available up to a maximum diameter of 13.5 mm. Consequently, patients with larger vessel diameters cannot be treated.

This study shows that endovascular repair of various IALs with a self-expandable Nitinol stent graft is a safe and effective procedure and should be considered as an additional treatment option in patients with this type of lesions.
